# Steroid-Induced Pancreatitis: Establishing an Accurate Association Poses a Challenge

**DOI:** 10.7759/cureus.9589

**Published:** 2020-08-06

**Authors:** Arun Minupuri, Roshni Patel, Fahad Alam, Manzoor Rather, Riaz H Baba

**Affiliations:** 1 Internal Medicine, Mercy Catholic Medical Center, Darby, USA

**Keywords:** steroids, acute pancreatitis, drug induced

## Abstract

The association between steroids and pancreatitis has been reported in the literature. However, due to its rarity, it can be challenging to make an early diagnosis. Hence, when diagnosing patients presenting with signs and symptoms of pancreatitis, there should be a high suspicion for medication-induced variants, after ruling out other common causes. In our report, we present the case of an individual with recurrent pancreatitis caused by the use of prednisone for musculoskeletal pain, the probable cause being steroids due to a high Naranjo score. The patient experienced clinical improvement with the resolution of pancreatitis after the steroids were discontinued.

## Introduction

Acute pancreatitis is defined as the inflammation of the pancreas; it is characterized by symptoms of abdominal pain radiating to the back, fever, nausea, and vomiting [1]. Common causes include gallstones, alcohol use, and hypertriglyceridemia. Drug-induced pancreatitis is very rare [1,2]. It accounts for just 0.1-2% of the total acute pancreatitis incidents [3]. Although it has a mortality rate of only <1%, the failure to identify medications that caused the condition can significantly delay patient care and lead to devastating results [3]. Management involves removing the offending agent, followed by continued supportive care [4]. In this report, we describe a rare case of recurrent pancreatitis caused by steroids.

## Case presentation

A 61-year-old Caucasian female with a past medical history of hyperlipidemia and pancreatitis presented to our institution with complaints of acute epigastric pain of one day's duration. The patient reported that her pain was similar to the one she had felt in a previous episode of pancreatitis around four years ago. At that time, gallstones, alcohol, electrolyte, metabolic, infectious, and autoimmune etiologies had been ruled out. She had been treated as a case of drug-induced pancreatitis due to rosuvastatin and had been discharged with instructions for outpatient gastroenterology follow-up.

On her present admission, she described epigastric pain associated with nausea and vomiting. Per patient, she had resumed her rosuvastatin medication one year prior with no complications or side effects. Her only new medication was a course of prednisone 10 mg/day for musculoskeletal pain, which was the exact same prednisone course she had been taking prior to her previous episode of acute pancreatitis. She had been on the current prednisone course for a week.

**Figure 1:**
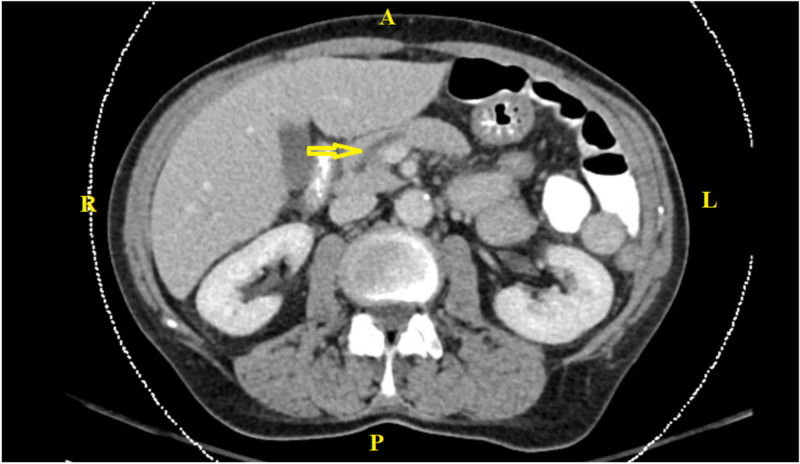
CT of the abdomen and pelvis with contrast The image shows subtle fat stranding along the pancreatic head and slightly prominent pancreatic duct, which can be seen with early interstitial edematous pancreatitis (arrow) CT: computed tomography

On admission, her vitals were significant for elevated blood pressure. Labs showed a lipase level of 169 mg/dL, triglyceride of 85 mg/dL, calcium of 8.9 mg/dL, ethyl alcohol level of <10 mg/dL, and negative hepatitis panel. Abdominal CT revealed subtle fat stranding along the pancreatic head and slightly prominent pancreatic duct, which can be seen with early interstitial edematous pancreatitis (Figure 1). Gastroenterology was consulted, and they suggested that it was likely due to steroid use. Her steroids were stopped, and her rosuvastatin was continued. The patient additionally received supportive care with IV fluids and analgesics. Her symptoms improved significantly within 48 hours, and she was discharged with instructions for outpatient follow-up with primary physician and gastroenterologist. The patient was advised to avoid steroid use, and she has had no complications since then.

## Discussion

Steroid-induced pancreatitis is a topic that is generally not well-studied or reported [3]. Steroids have many known side effects through their inhibition of inflammatory mediators and prostaglandins [5]. It has been suggested that steroids can affect the pancreas by increasing the viscosity of pancreatic secretions and delaying the emptying [6]. Acute pancreatitis is most commonly caused by gallstones, alcohol use, hypercalcemia, hypertriglyceridemia and familial, autoimmune, and post-surgical issues [7]. But in cases where all of these are ruled out, medications in the form of steroids can be the most likely cause [7].

Pancreatitis is a rare side effect of steroid use [8]. Reports that effectively isolate steroids as the inciting agent are very rare, and a delay in the proper diagnosis of the condition can lead to complications and prolonged hospital stay [3]. Complications include local and systemic ones, which can present as pancreatic pseudocyst, pancreatic necrosis and infection, chronic pancreatitis, and multiorgan failure [9].

In this case, this patient presented with pancreatitis on two separate occasions, both occurring after steroid exposure. Given a Naranjo score of 7, it is most likely that her pancreatitis was due to steroid use [10]. On both presentations, the patient showed immediate clinical improvement after steroids were discontinued. As steroids are a commonly used medication, it is important to consider it as a possible cause of pancreatitis in cases where other causes have been ruled out. 

## Conclusions

When caring for patients presenting with signs and symptoms of pancreatitis, clinicians should maintain a high suspicion for medication-induced (in the form of steroids) variants, after ruling out common causes such as gallstones and alcohol use. A delay in diagnosis can lead to complications and prolonged hospital stay. Prompt cessation of the offending medication can lead to clinical improvement with resolution of pancreatitis.
